# Histopathological Diagnosis of a Case of Mucin-Secreting Adenocarcinoma of the Appendix

**DOI:** 10.7759/cureus.63435

**Published:** 2024-06-29

**Authors:** Rishika Bhatnagar, K.M. Hiwale, Snehlata Hingway

**Affiliations:** 1 Pathology, Jawaharlal Nehru Medical College, Datta Meghe Institute of Higher Education & Research, Wardha, IND

**Keywords:** carcinoma of the appendix, mucinus adenocarcinoma, appendiceal mucinous neoplasm, pseudomyxoma peritonei, low grade appendiceal mucinous neoplasm

## Abstract

Mucin-secreting adenocarcinoma of the appendix is a very rare, slow-growing, mucin-producing epithelial neoplasm of the appendix. It is usually found accidentally in an appendicectomy specimen with the presentation of acute appendicitis in most patients or when there is a rupture of the primary tumor with the mucin spreading along with the tumor cells in the entire peritoneal cavity. Here we describe a case of low-grade (well-differentiated) mucin-secreting adenocarcinoma in the appendix. A 48-year-old female presented with complaints of abdominal distension with no other complaints of fever, pain, or breathlessness. Carcinoembryonic antigen levels were 44.8 ng/mL. Cytoreduction surgery of bilateral ovaries was done. The final histopathological diagnosis was reported as low-grade (well-differentiated) mucin-secreting adenocarcinoma of the appendix staged at pT4b pNx pM1c. Pseudomyxoma peritonei is a very feared complication and also, at times, the only presenting symptom where there is an accumulation of mucin in the intra-abdominal cavity due to the spread of mucin-secreting cells, which in turn causes an increase in the abdominal girth along with discomfort for the patient. The mainstay of treatment remains cytoreductive surgery along with hyperthermic intraperitoneal chemotherapy.

## Introduction

Mucin-secreting adenocarcinoma of the appendix is a very rare, slow-growing, mucin-producing epithelial neoplasm of the appendix that has an incidence of 0.12 cases per 1,000,000 people [1]. It is estimated to be around 0.2-0.3% of all appendicectomy specimens [2]. Out of these, histologically, 65% of the tumors are neuroendocrine in origin, while only about 20% are adenocarcinomas that include mucinous, nonmucinous, and signet ring types. Appendiceal mucinous tumors can cause in the peritoneum a phenomenon known as pseudomyxoma peritonei (PMP), which is an accumulation of gelatinous mucin (mucinous ascites). In most cases, appendiceal malignancy is an incidental finding, with no apparent symptoms in the patient that raise suspicion of a preexisting neoplastic etiology. However, due to the presence of PMP, there may be an increase in the abdominal girth of the patient, which was seen in our patient with no other symptoms. Also, the patient can present as a case of acute appendicitis, with malignancy being detected in a later histopathological evaluation [3]. Given the low rate of incidence of mucin-secreting adenocarcinoma in the appendix, i.e., 0.12 cases per 1,000,000 people, with only about 0.2-0.3% of appendectomy specimens to be reported to have this diagnosis on histopathology, witnessing this case is rare and a golden opportunity to be a part of the process of its final diagnosis. Hence, we report this case of a 48-year-old female who presented with abdominal distention and no other signs or symptoms and was otherwise skeptical of any neoplastic etiology of the gastrointestinal tract. She was eventually diagnosed and treated as a case of mucin-secreting adenocarcinoma of the appendix with PMP.

## Case presentation

**Figure 1:**
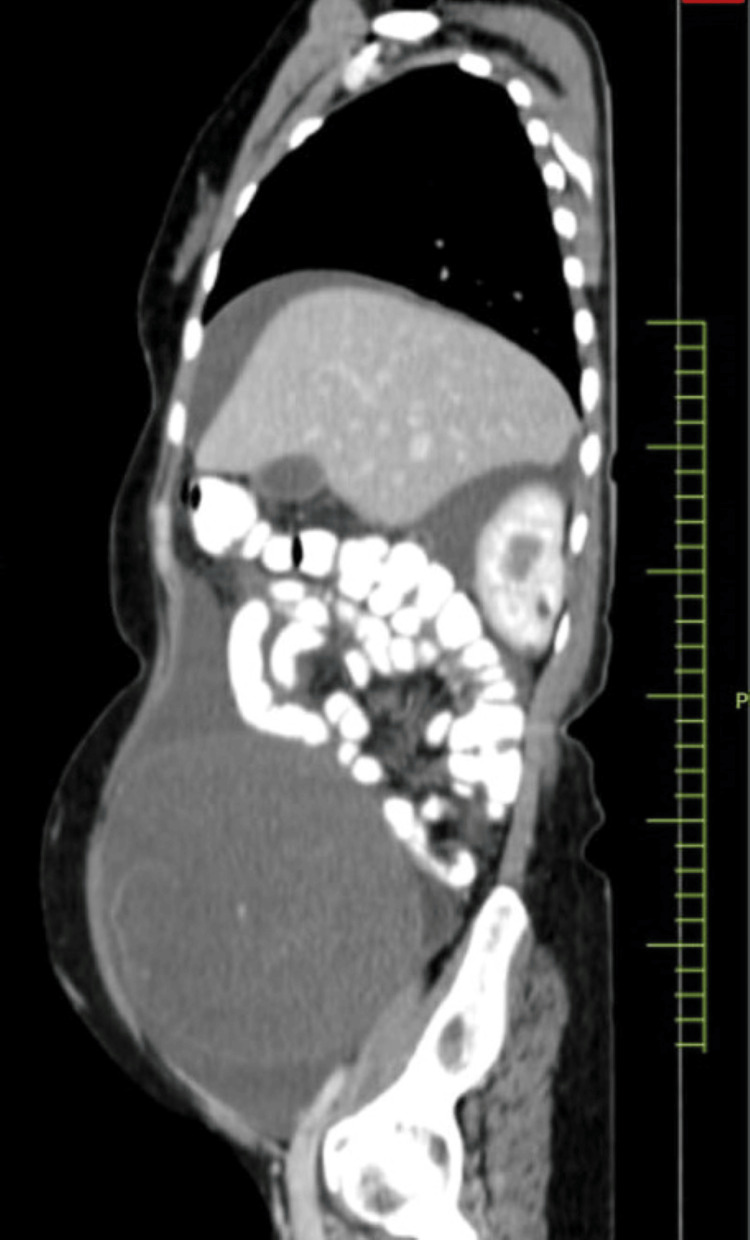
CT image shows a large, well-defined, solid cystic lesion in the pelvis in the midline, extending up to the level of the umbilicus and measuring 185 × 141 × 201 mm

**Figure 2:**
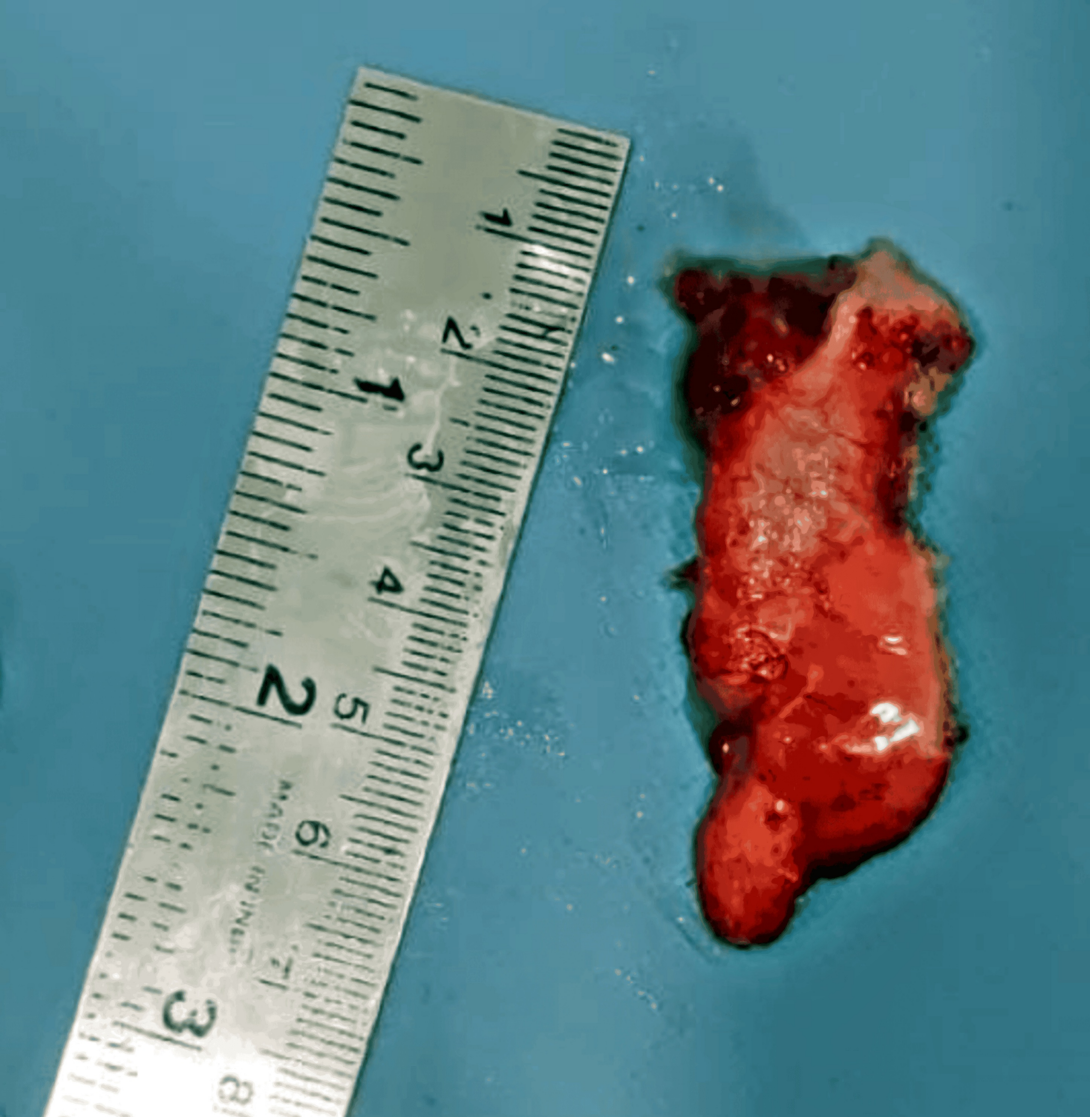
Gross image of an appendicectomy specimen, measuring 3.2 cm in length

**Figure 3:**
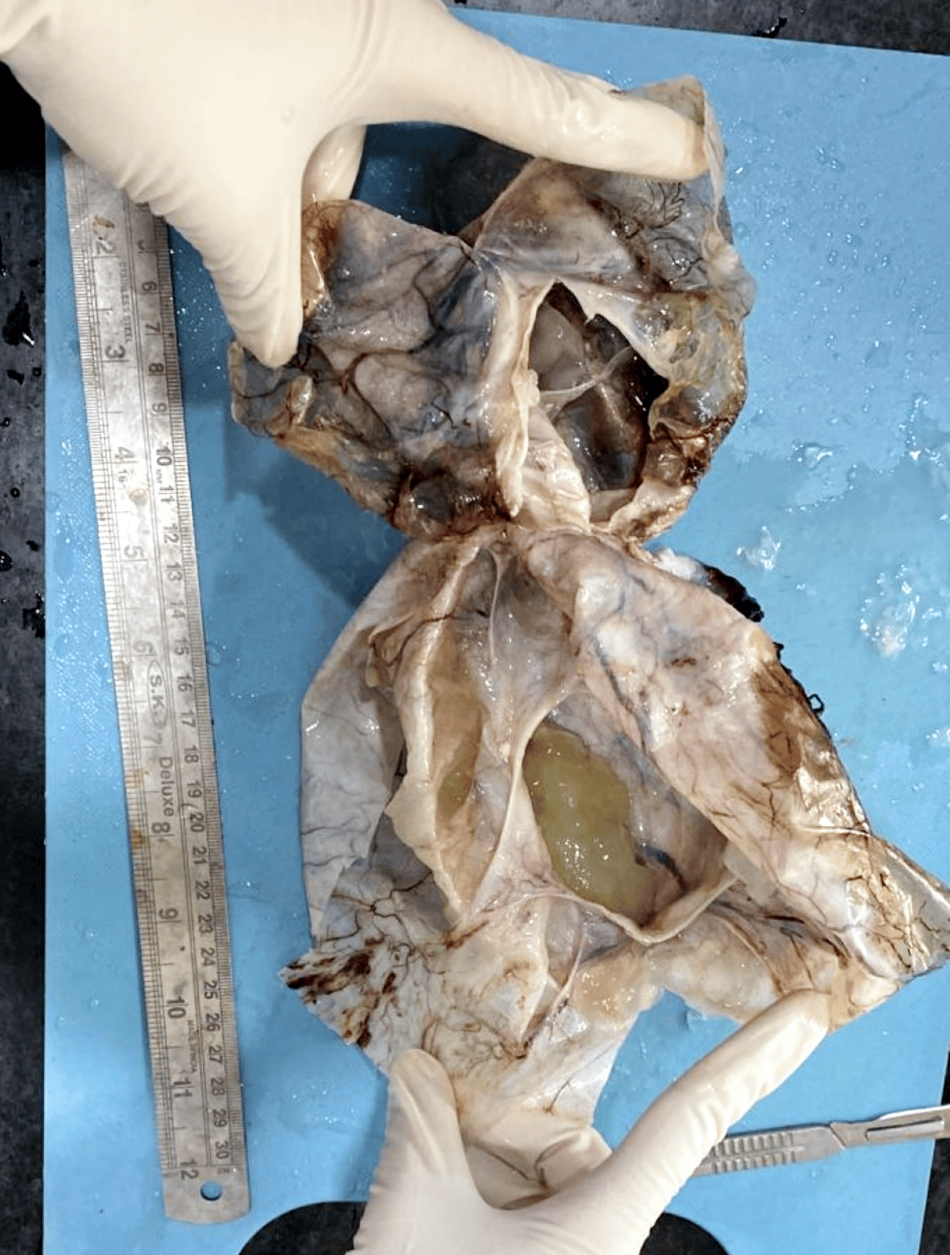
Bilateral ovaries with large mucin-filled cysts, from which yellowish-white, jelly-like mucinous fluid oozed out

**Figure 4:**
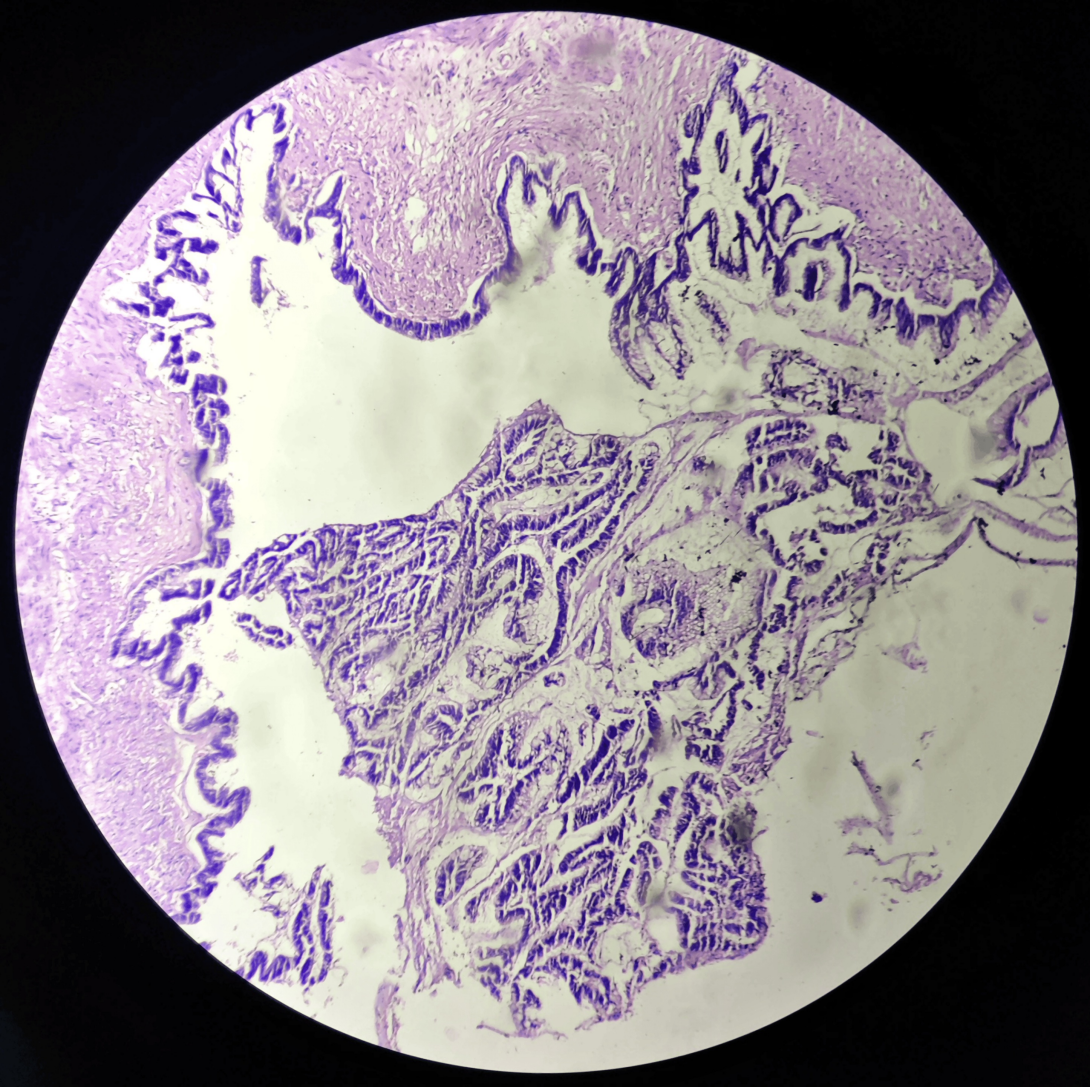
H&E section showing abundant extracellular mucin with irregular glands floating in it, along with areas of loss of lamina propria and atrophy of the muscularis mucosae

A 48-year-old female presented with complaints of abdominal distention and no other complaints of fever or breathlessness. There was no tenderness on palpation in the right lower quadrant. She had normal vitals on arrival. The patient had a history of a vaginal hysterectomy in 2016 for uterine prolapse. A CT scan was done, which showed evidence of a large, well-defined, solid cystic lesion in the pelvis in the midline, extending up to the level of the umbilicus, suggesting the presence of ascitic fluid and large ovaries. The lesion measured 185 × 141 × 201 mm in size (Figure 1). The imaging suggested the possibility of a neoplastic etiology - mucinous cystadenocarcinoma arising from the ovary. Further, carcinoembryonic antigen levels were done, which turned out to be 44.8 ng/mL. Cytoreduction surgery of bilateral ovaries was planned via an abdominal approach. A resected specimen of the omentum with a falciform ligament and an appendix was received in pathology for further evaluation. The appendectomy specimen measured 3.2 cm in length (Figure 2). It was enlarged and dilated. On the cut section, a thickening of the wall was identified across the entire lumen of the appendix. Cystic growth measuring 1 × 1 × 0.3 cm was seen on the proximal part of the appendix. Bilateral ovaries were also received that had huge mucin-filled cysts (Figure 3). On the cut section, jelly-like mucinous fluid oozed out (yellowish-white). On histopathology, sections showed abundant extracellular mucin with irregular glands floating in it. It also showed areas with loss of lamina propria and atrophy of muscularis mucosae (Figure 4). The final histopathological diagnosis was reported as low-grade (well-differentiated) mucin-secreting adenocarcinoma of the appendix staged at pT4b pNx pM1c. Postoperatively, the patient was stable and maintained her vitals. She visited the outpatient clinic for follow-up every three months. She did not have complaints, and the scar was healing adequately at the six-month mark.

## Discussion

Neoplasms with an appendix as the primary source of origin are a very rare entity to come across. They can arise from two sources, namely the nonepithelial source of origin, i.e., neuroendocrine cells, and the epithelial source of origin, which are the cells lining the appendix, which secrete mucin, a jelly-like substance that has protective functions in the appendix, stomach, and intestines. When there is a spread of these mucin-secreting cells into the abdominal cavity, there is an accumulation of this mucin in the intra-abdominal cavity; this condition is called PMP. This leads to mucinous ascites in the patient, causing increased abdominal girth, which at times is the only presenting symptom (as in the case of our patient). Other types of epithelial tumors include goblet cell adenocarcinoma, signet ring cell adenocarcinoma, and colonic type adenocarcinoma (which is nonmucinous) [4]. Mucin-secreting adenocarcinoma of the appendix is a very rare, slow-growing, mucin-producing epithelial neoplasm of the appendix that has an incidence of 0.12 cases per 1,000,000 people [1]. It is estimated to be around 0.2-0.3% of all appendicectomy specimens [2].

In 2010 [5], WHO classified mucinous neoplasms in the following three categories with various differentiating features based on the extent of invasion, presence of extracellular mucin, presence or absence of PMP, and survival rate: mucinous adenoma, low-grade appendiceal neoplasm, and mucinous adenocarcinoma.

In most cases, it is an incidental finding in the patient either intraoperatively (which is mostly ambiguous with no confident approach toward the diagnosis) or on histopathology, with no obvious symptoms that raise suspicion of any sort of neoplastic etiology. Initially, the patients do not have any obvious symptoms or may present with features of acute appendicitis, such as acute abdominal pain and fever. However, in asymptomatic patients, the spread of mucin-secreting cells beyond the boundaries of the appendix leads to the accumulation of mucin in the abdominal cavity, causing abdominal distention, eventually leading to dyspepsia, early satiety, and a lack of hunger due to the pressure effect [4]. Metastasis outside of the peritoneal cavity is very rare [6]. The patient may also mimic a scenario of acute presentation with pain in the right lower quadrant and fever. In such cases, an emergency appendicectomy is done, wherein cystic dilatation of the appendix may be seen and is later confirmed on histopathology.

Radiological investigations such as ultrasonography and CT scans can be done to look for the presence of any mass-like etiology. Cystic dilatation of the appendix can be seen in CT scans [7]. However, the confirmatory diagnosis is made on histopathology with the presence of abundant extracellular mucin with irregular glands floating in it. There are elongated nuclei with low-grade nuclear atypia in cases of low-grade appendiceal mucinous neoplasm and compressed nuclei with high-grade nuclear atypia in cases of high-grade appendiceal mucinous neoplasm. Also, there are areas with loss of lamina propria and atrophy of muscularis mucosae. Calcifications, along with internal septations and periappendiceal fat, can also be visualized [8].

Tumor-node-metastasis staging is done as per the American Joint Committee on Cancer (AJCC) guidelines in accordance with the 7th Edition.

The mainstay of treatment remains cytoreductive surgery (CRS) along with hyperthermic intraperitoneal chemotherapy (HIPEC). The mean survival rate of low-grade neoplasms is 100% when they are confined to the appendix itself [7].

## Conclusions

Mucin-secreting adenocarcinoma is a very rare neoplasm of the appendix, which is a sluggishly growing mucin-producing tumor of the epithelial cells with an infrequent incidence of 0.12 cases per 1,000,000 people. Among all the appendicectomy specimens received, only about 0.2-0.3% were found to be mucin-secreting adenocarcinomas. Usually, it is diagnosed accidentally, either with patients presenting with symptoms of acute appendicitis or intraoperatively, with a final diagnosis based on histopathological evidence, since most of the patients do not give away any pointers. PMP is a very feared complication and, at times, the only presenting symptom. In this case, there is an accumulation of mucin in the intra-abdominal cavity due to the spread of mucin-secreting cells, which in turn causes an increase in the abdominal girth along with discomfort for the patient. The mainstay of treatment remains CRS, along with HIPEC.

To have encountered such a golden case and the comprehensive efforts taken to come to a definitive diagnosis, we hope to report an exquisite case with a classic presentation that would further be useful for fellow physicians and pathologists in understanding the rarity of such a neoplasm with an apt definitive diagnosis.
